# Relationships between Meteorological Parameters and Particulate Matter in Mae Hong Son Province, Thailand

**DOI:** 10.3390/ijerph15122801

**Published:** 2018-12-10

**Authors:** Wissanupong Kliengchuay, Aronrag Cooper Meeyai, Suwalee Worakhunpiset, Kraichat Tantrakarnapa

**Affiliations:** 1 Department of Social and Environmental Medicine, Faculty of Tropical Medicine, Mahidol University, Bangkok 10400, Thailand; wissanupong.k@gmail.com (W.K.); suwalee.wor@mahidol.ac.th (S.W.); 2 Department of Epidemiology, Faculty of Public Health, Mahidol University, Bangkok 10400, Thailand; a.meeyai@gmail.com

**Keywords:** particulate matter, meteorological parameters, Mae Hong Son Province

## Abstract

Meteorological parameters play an important role in determining the prevalence of ambient particulate matter (PM) in the upper north of Thailand. Mae Hong Son is a province located in this region and which borders Myanmar. This study aimed to determine the relationships between meteorological parameters and ambient concentrations of particulate matter less than 10 µm in diameter (PM_10_) in Mae Hong Son. Parameters were measured at an air quality monitoring station, and consisted of PM_10_, carbon monoxide (CO), ozone (O_3_), and meteorological factors, including temperature, rainfall, pressure, wind speed, wind direction, and relative humidity (RH). Nine years (2009–2017) of pollution and climate data obtained from the Thai Pollution Control Department (PCD) were used for analysis. The results of this study indicate that PM_10_ is influenced by meteorological parameters; high concentration occurred during the dry season and northeastern monsoon seasons. Maximum concentrations were always observed in March. The PM_10_ concentrations were significantly related to CO and O_3_ concentrations and to RH, giving correlation coefficients of 0.73, 0.39, and −0.37, respectively (*p*-value < 0.001). Additionally, the hourly PM_10_ concentration fluctuated within each day. In general, it was found that the reporting of daily concentrations might be best suited to public announcements and presentations. Hourly concentrations are recommended for public declarations that might be useful for warning citizens and organizations about air pollution. Our findings could be used to improve the understanding of PM_10_ concentration patterns in Mae Hong Son and provide information to better air pollution measures and establish a warning system for the province.

## 1. Introduction

Air pollution is a critical issue in both developed and developing countries [1,2]. Types of air pollution vary from place to place and depend upon human activity, topography, and other factors. Meteorological factors influence air pollution levels; wind direction and wind speed, for example, are key factors guiding air movement [3,4,5]. Other meteorological factors also play an important role in the dispersion of air pollution, and their influence can differ by season, and by day and night. [6]. Many researchers have reported that the concentration of air pollutants varies depending on meteorological factors [4], the source of pollutants, and local topography [7]. Particulate matter less than 10 µm in diameter (PM_10_) concentrations from Athens and Birmingham have been found to be significantly correlated with other pollutants and meteorological parameters, namely, nitrous oxides (NO_x_), carbon monoxide (CO), and solar radiation [8]. Negative correlations between PM_10_ concentration and the secondary pollutant ozone (O_3_), wind speed (WS), and precipitation have been observed [9]. PM_10_ has also been found to be inversely correlated with temperature, and relative humidity (RH) [10]. Correlations between PM_10_ and WS have been observed as negative in the summer and fall, but positive in winter [11].

Haze episodes have been observed annually in the upper north of Thailand. Normally, PM_10_ is used as an indicator to determine if a haze episode is occurring, that is, if the daily average concentration of PM_10_ exceeds the standard concentration (120 ppm) as set by the Thai Pollution Control Department (PCD). The influence of meteorological parameters on ambient air pollution has been confirmed [12]. Many countries use the concentration of particulate matter with a diameter of 2.5 micrometers or less (PM_2.5_) as a health indicator, but the existence of PM_2.5_ monitoring stations in Thailand is limited to big cities like Bangkok and Chiang Mai Province. One study has confirmed that atmospheric pollutants in Chiang Mai Province are directly related to meteorological conditions such as the atmospheric cycle, and indirectly to others, resulting in increased burning during the dry season [13]. The transboundary effect of air pollution from neighboring countries, facilitated by the long range transport phenomenon, has been found as another influencing factor [14]. Haze episodes affect not only human health but also socio-economic factors such as the predominance of accidents, and transportation and tourism [1,15]. Socio-economic impacts such as decreasing tourist numbers, and the cancelation of bookings for hotels and related tourist service industries have been observed [16]. The critical issue is impacts on human health caused by particulate matter exposure; hospitalization and treatment have sometimes resulted. Human health impacts consisted of both diseases related to short-term exposure and chronic diseases resulting from long term exposure [15].

Central and local governments have increased efforts to mitigate these problems, including attempting to reduce PM_10_ concentrations. Many approaches have been employed to reduce air pollution levels. For example, legislation to stop biomass burning after the harvesting period, forest fire controls, and other efforts have been introduced [17]. Policies and mitigation measures have been implemented and strengthened in big cities such as Chiang Mai, but pollution has still not been eliminated. Law enforcement groups from local municipalities and the private sector have joined forces to reduce pollution levels [16]. Mae Hong Son Province (a small mountainous tourist area located close to the border between Thailand and Myanmar) suffers from various air pollution factors, such as the transboundary effect, forest fires, and biomass burning. An understanding of factors influencing haze episodes in this province will aid the ability of stakeholders (including local/national policy makers and the private sector) to design air pollution mitigation methods [18] and improve health. For this reason, this study aimed to delineate the relationships between meteorological parameters and ambient PM_10_ concentration (comprising concentrations both local and transboundary in origin) in Mae Hong Son Province.

## 2. Methods

### 2.1. Description of the Study Area

**Figure 1:**
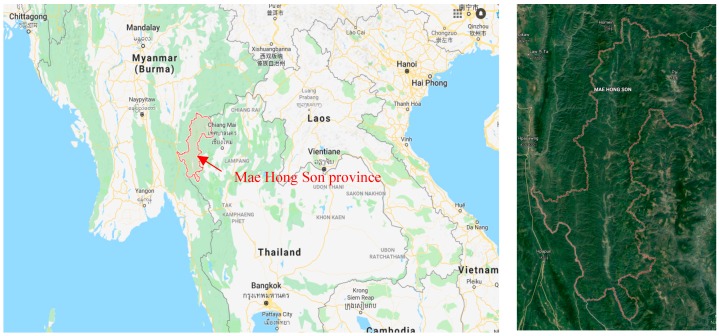
Location of Mae Hong Son, Thailand [19].

Mae Hong Son is located in the upper north of Thailand on the country’s western border (Figure 1). Neighboring provinces include the Shan State of Myanmar, Chiang Mai, and Tak (clockwise from north to south). The Kayin and Kayah States of Myanmar are to the west. Mae Hong Son remains a popular tourist attraction, especially for those who appreciate its natural features and the cultural variety of local ethnic groups. The province has been called “the city of three mists” due to its proximity to several mountainous areas which cause it to be frequently covered with mist. Most land within the province is forested, while the main occupation of its inhabitants is agriculture. Mae Hong Son is located between the latitudes 17°38′ N and 19°48′ N, and longitudes 97°20′ E to 98°39′ E. It has an area of 12,681.26 km^2^. A topographical map of Mae Hong Son is given in Figure 1.

### 2.2. Data Measurement and Analysis

The meteorological parameters of RH, temperature, rainfall, WS, and wind direction, as well as concentrations of common air pollutants such as PM_10_, O_3_, and CO are measured and recorded at hourly intervals by the PCD, a sub-organization of Thailand’s Ministry of Natural Resources and Environment. Nine years of data (2009–2017) obtained from the PCD were cleaned as the first step in this study. R Software (R-Studio, R Foundation for Statistical Computing, Vienna, Austria) was used as a tool for data analysis. R is an open-source computer programming system which is swiftly gaining use across various disciplines including Environmental Health [20]. It is an interpreted language that offers excellent interactive analysis capabilities and is ideal for the rapid development of statistical and data analysis applications [21]. The “open air” package was employed in this study for statistical analysis and graph generation, as it can be used for air pollution analysis and other statistical modeling. The statistical methods for data analysis used included descriptive statistics such as mean, standard deviation, quartile, and Spearman’s correlation. The latter was employed to determine correlation values between the relevant parameters.

HYSPLIT4 Software (NOAA, Silver Spring, MD, United States) and the Ready website were used to compute simple air parcel trajectories and construct complex dispersion and deposition simulations [22]. The focus of this study was on the back trajectories of air parcels detected in Mae Hong Son. The back trajectories provided the Lagrangian path of the air parcels within a chosen time scale, which could then be used to recognize the source of pollutants that fell within the track of the back trajectories.

Meteorological parameter and pollutant concentration data were collected at hourly intervals to obtain an average daily concentration that could be compared to the PCD ambient air quality standard. Daily average meteorological parameters quantities and pollutant concentrations were calculated only when valid data were available for more than 20 h over the course of that day. The correlation of meteorological data and particulate matter (PM) concentration was performed using the Spearman-rank method.

In addition, researchers performed on-site measurement of both PM_2.5_ and PM_10_ at two stations during a haze episode for three consecutive days in April 2017. The membrane filters for PM_2.5_ and PM_10_ measurement were PTFE (Polytetrafluoroethylene) sizes 25 mm and 37 mm, respectively. Quality assurance/control was performed for the entire air collection and measurement process.

## 3. Results and Discussion

The characteristics of air pollution and meteorological data from 2009 to 2017 in this province are summarized in Table 1; 7.15% of PM_10_ data were missing according to the monitoring system. Data indicated that the maximum 1 h concentration of PM_10_ was 726 ppm in 2013. Daily PM_10_ concentrations greater than the standard level of 120 ppm totaled 9.3%. The daily average concentration for the period 2009 to 2017 was 42.7 ppm, with a daily maximum concentration of 309.2 ppm. Other pollutants were within acceptable levels according to the PCD standard. The annual average concentration of PM_10_ was 43.02 ppm. Mae Hong Son Province also experienced high temperatures from 2009 to 2017, exhibiting extreme highs during the dry season with a maximum temperature of 45.5 °C.

The greatest PM_10_ concentration obtained from monitored data was found to be 726 ppm, whereas the daily average concentration was 309.2 ppm. This means that PM_10_ concentrations fluctuated over the course of a whole day. The daily average concentration can be used to illustrate the average concentration for a 24 h period. However, the maximum concentration can be detected at any time of the day. It is recommended that hourly concentration data be presented to vulnerable groups who are sensitive to higher concentrations of pollutants. This would enable them to better prepare themselves, particularly for long outdoor journeys. Concentrations may be influenced by climate-related factors.

### 3.1. Onsite PM Measurement

PM_2.5_ and PM_10_ were measured over the period 7–9 April 2017, revealing a daily average concentration and standard deviation for PM_2.5_ and PM_10_ of 138.8 ± 22.7 and 171.5 ± 20.6 ppm, respectively. The ratio of PM_2.5_ to PM_10_ concentrations ranged from 0.78 to 0.83, with an average value of 0.81 and a standard deviation of 0.03. The maximum concentration was 1.4 times the standard concentration recommended by the PCD (120 ppm) [23]. The average concentration measured over the same three-day period by the PCD at a monitoring station near our location was 125.4 ppm, with a standard deviation of 45.1 ppm. Our measured concentrations were 1.4 times higher than those recorded by the PCD for the same period (7–9 April 2017). This might be due to differences in air volume and other factors, such as wind direction [23].

The PCD do not currently measure PM_2.5_ concentrations in Mae Hong Son Province. However measurements from this study indicate that daily PM_2.5_ concentrations are high compared to the PCD standard and World Health Organisation (WHO) guidelines. The average daily concentration and standard deviation of PM_2.5_ from 7–9 April 2017 was 138.8 ± 22.7 ppm. This was approximately 2.7 times the PCD standard (50 ppm). In addition, we found the ratio of PM_2.5_ to PM_10_ concentrations was 0.83. According to data collected by the PCD for the same period, the maximum daily concentration of PM_10_ was 309 ppm. Based on this value, the maximum daily concentration of PM_2.5_ might be approximated as 256.47 ppm, which is equivalent to 5.1 times the PCD standard. This concentration level has been classified as hazardous to all people. A PM_2.5_ monitoring system is evidently needed in Mae Hong Son Province. Furthermore, it would be favorable for a predictive system giving air quality predictions up to three days in advance to be developed, as this could be used as a warning system for both residents and tourists in this province [16].

### 3.2. Time Series

**Figure 2:**
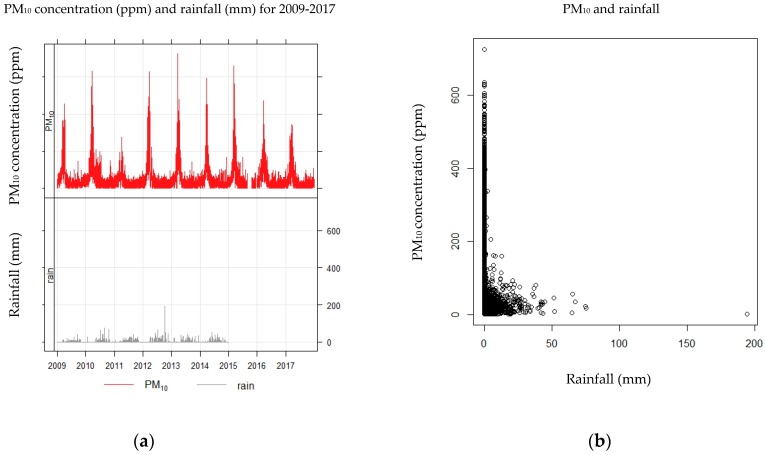
(**a**) PM_10_ concentration(ppm) and rainfall (mm) for 2009-2017, (**b**) PM_10_ and rainfall.

When considering PM_10_ concentration levels over the nine-year period examined in this study, the greatest PM_10_ concentrations were seen for the year 2013, whereas a much lower PM_10_ concentrations were observed for the year 2011. This is shown in Figure 2a. Variations in PM_10_ concentration within each annual period were similar for every year. Peak concentrations were always found to occur in the dry season, particularly in March. 

A time series analysis was undertaken on data gathered for the 2009–2017 period studied, and it was found that the smallest PM_10_ concentrations were observed in 2011. The reason for these low concentrations was a devastating flood in Thailand. The amount of rainfall in 2011 was 1686.1 mm, which was higher than for any other year, as indicated by Table 2. In addition, the time series plot between PM_10_ concentration and rainfall was consistent for the period 2009–2017. Rainfall data for the years 2015–2017 are not available because of monitoring equipment failure during this time. Figure 2b indicates that PM_10_ concentrations and rainfall levels were significantly correlated (*p*-value < 0.001). At lower rainfall levels, higher concentrations of PM_10_ were observed. High concentrations of particulate matter were always present in the dry season, that is, between February and April.

**Figure 3:**
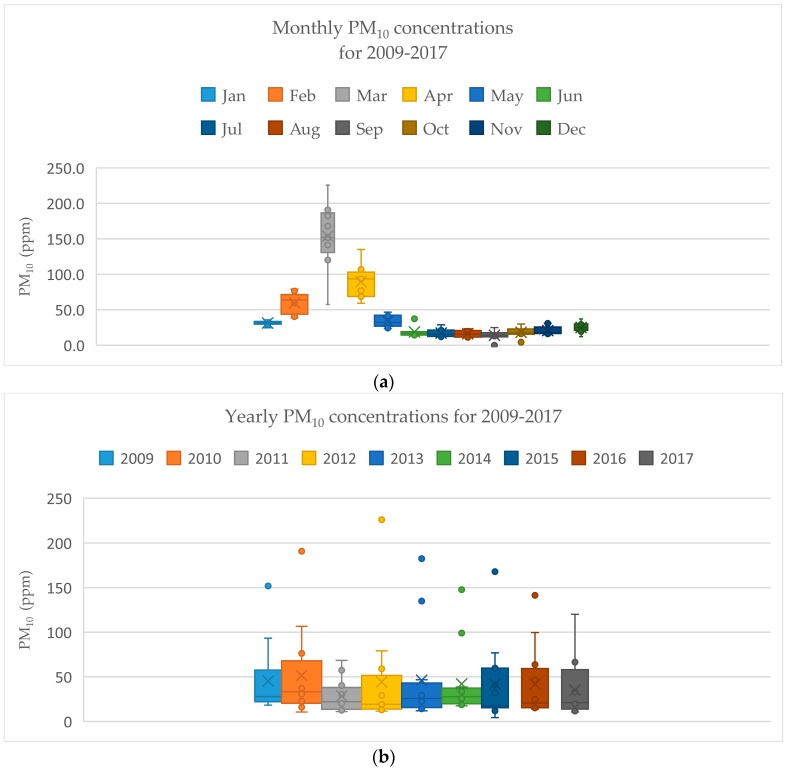
Monthly (**a**) and yearly (**b**) PM_10_ concentration in Mae Hong Son Province for 2009–2017.

Monthly and yearly PM_10_ concentrations were also analyzed, as illustrated in Figure 3a,b. Regarding monthly concentration levels, PM_10_ concentrations were clearly greatest in March. With regard to yearly PM_10_ concentrations, the average PM_10_ concentration was detected in 2011. However, PM_10_ concentrations did not fluctuate significantly between each year as illustrated in Figure 3b.

### 3.3. Correlation between PM_10_ and Other Pollutants and Meteorological Parameters

**Figure 4:**
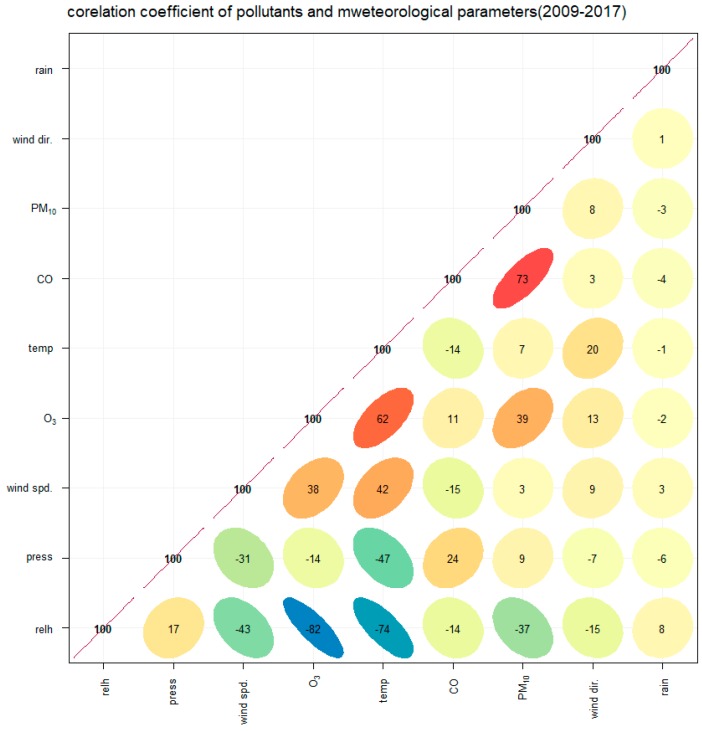
Correlation coeeficients of pollutants and meteorological parameters for 2009–2017.

Spearman rank correlations between PM_10_ concentrations and levels of other air pollutants revealed significant correlations between PM_10_ concentrations and concentrations of CO and O_3_. These pollutants had correlation coefficients of 0.73 and 0.39, respectively, as indicated by Figure 4 (*p*-value < 0.001). 

With regard to the correlation of PM_10_ concentrations and meteorological parameters, PM_10_ was negatively correlated with RH, producing a correlation coefficient of −0.36 (*p*-value < 0.001). This result matches findings from Jayamurigan et al. and Zhao et al. [25,26]. O_3_ was the second most major pollutant and its concentration levels were positively associated with temperature, giving a correlation coefficient of 0.62. O_3_ was also negatively associated with RH (r = −0.82), as has been indicated by Atkinson [27]. H. Zhang et al. have also found similar correlations between RH and PM_10_ concentrations in Shanghai and Guangshou in China [11].

### 3.4. Seasonal Variation

**Figure 5:**
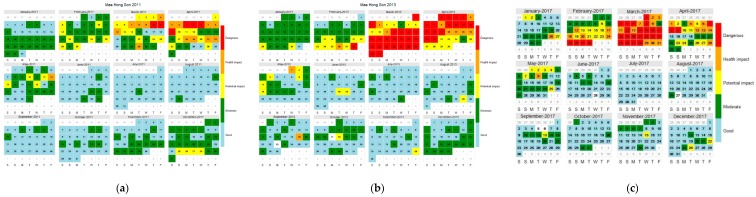
Calendar presentations of daily PM_10_ concentrations in Mae Hong Son Province for 2011 (**a**), 2013 (**b**), and 2017 (**c**).

Normally, Thailand’s three seasons are classified as summer (March–May), rainy (June–October), and winter, or the cold season (November–February). As seen in the time series produced by this study, PM_10_ concentrations gradually increased in the early part of each year, starting in January and reaching their maximum levels by March. During the nine years monitored here, PM_10_ concentrations were lowest in 2011, and the maximum concentration level was detected in March 2013. Monthly variations in PM_10_ concentrations for 2011 and 2013 were chosen to be presented in an accessible calendar format, as shown in Figure 5. The color indicates the level of PM_10_ concentration and its potential health impacts according to the PCD standard. The classification of PM_10_ concentration levels and health impacts consisted of five categories. These were (1) good quality (PM_10_ < 50 ppm), (2) moderate air quality (51 ppm < PM_10_ < 80 ppm), (3) potentially harmful (80 ppm < PM_10_ < 120 ppm), (4) harmful (120 ppm < PM_10_ < 180 ppm), and (5) hazardous (PM_10_ > 180 ppm). The colors were used as a communication device—blue meant good, green meant moderate, yellow meant potentially harmful, orange meant harmful, and red meant hazardous. This figure presents similar results to the time series, as hazardous and harmful levels were again observed to occur in March and April, that is, during the summer season. PM_10_ concentrations nearly reached critical levels in March and April because they were greater than the PCD standard of 120 ppm [23].

**Figure 6:**
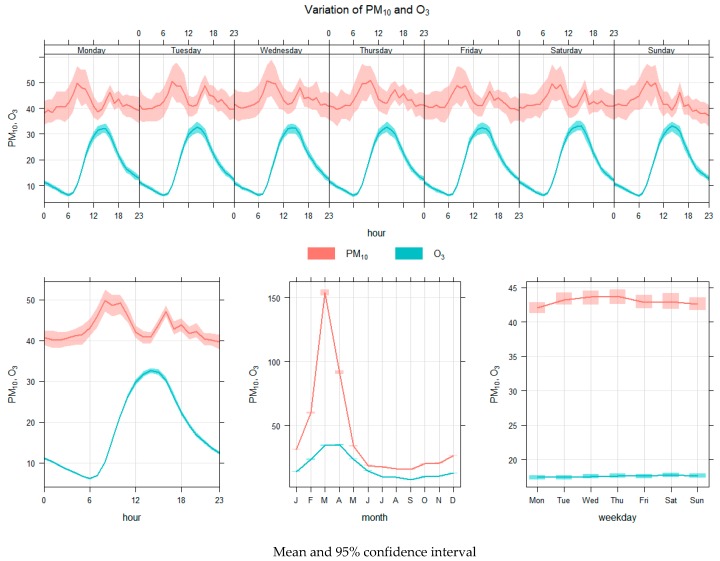
Variations in PM_10_ and O_3_ concentrations over hourly, daily, and monthly periods.

**Figure 7:**
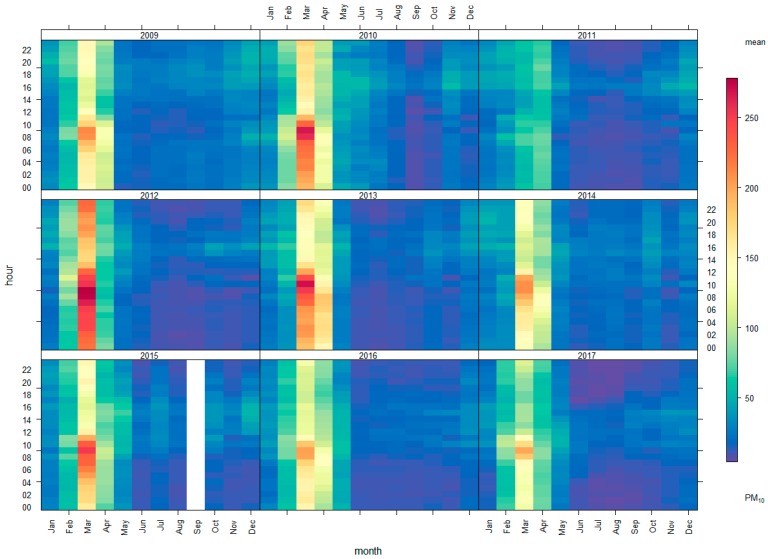
Variations in PM_10_ concentrations within each year, by hour and month.

PM_10_ concentrations varied for each period of the day. Two peaks were observed each day in the morning, from 7 to 10 am, and in the late afternoon, from 4 to 5 pm. By contrast, O_3_ concentrations peaked only once a day in the afternoon. The variation of both pollutants by date did not differ. The PM_10_ and O_3_ concentration patterns were similar, with a high concentration in March and April, and decreasing concentrations from May to November during the rainy season, as illustrated by Figure 6. Annual time variations were similar, with a high concentration in the morning. The greatest PM_10_ concentration at night was in the range of good to moderate, as indicated by its blue, green, and yellow coloring (Figure 7). With regard to the actual measurements from 7–9 April 2017, the average concentration was 138.5 ppm, which was similar to the data recorded by the PCD. Red was used for this concentration, as indicated in Figure 5c.

### 3.5. Wind Parameters

**Figure 8:**
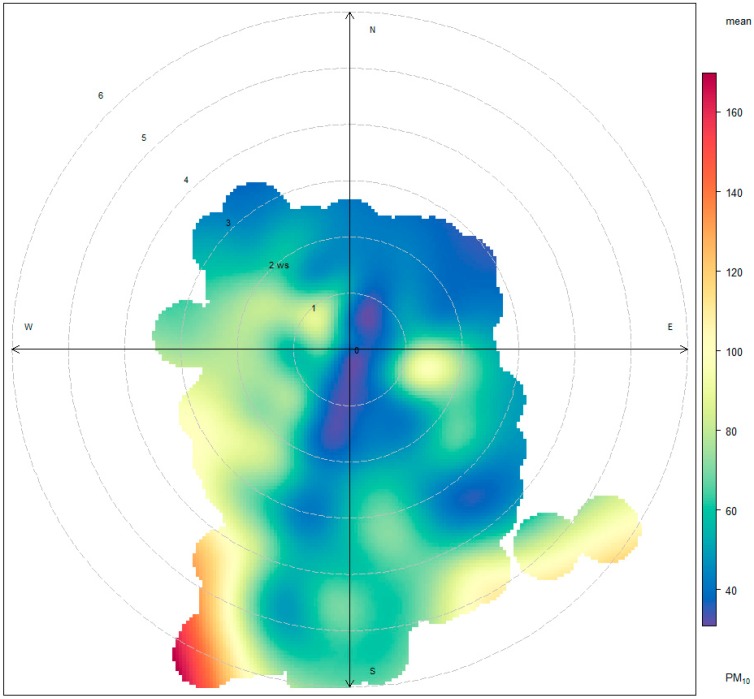
Mean concentrations of PM_10_ in relation to wind direction and speed.

The wind direction and WS were also observed as parameters that influenced PM_10_ concentration. Wind direction is an important parameter affecting PM. Wind from different directions transports different amounts of pollutants [28]. For the entirety of this study, high PM_10_ concentrations were generated when the wind was blowing from the north or northeast. Figure 8 indicates high concentrations using orange to red coloring, with PM travelling in a south-westerly direction, and with a WS of over 4 m/s. This indicates that wind blowing from the north or northeast during the northeastern monsoon season serves as an influencing factor on PM_10_ concentration levels.

The back trajectory model was also investigated using HYSPLIT4 software. A trajectory model can be used to calculate the position of the air being sampled backward in time from the receptor site from various starting times throughout the sampling interval. This study analyzed backward trajectories for 72 h during the observed haze period. The trajectories are presented as a sequence of latitude and longitude values within Mae Hong Son Municipality with the endpoint representing each designed time interval being modeled [29]. This model was used to calculate the air mass backward trajectories for those days when PM_10_ concentrations were monitored.

**Figure 9:**
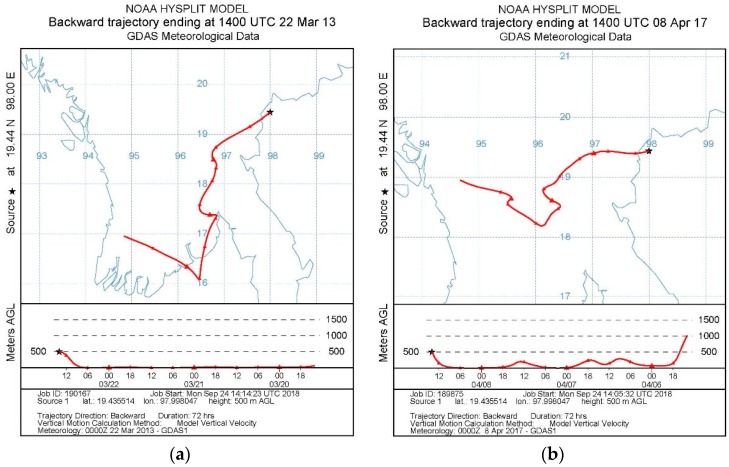
Calculated back trajectories for the 72 h period starting 24 March 2013 (**a**) and ending 8 April 2017 (**b**) at Mae Hong Son Pollution Control Department (PCD) Monitoring Station.

Weather data were obtained from the NOAA (National Oceanic and Atmospheric Administration) website by specifying the location of the study area. This study used the location of the weather monitoring station in Mae Hong Son. During the haze episode, the wind was always from the west and northwest, as indicated by Figure 9. This was similar to the results of a study conducted in Chiang Mai Province, which borders Mae Hong Son Province [30]. One related study indicated that more than 50% of the wind blew from the west and northwest. PM_10_ was seen to be not only generated locally but was dispersed from neighboring regions. The potential sources of PM_10_ might be biomass burning, local activities, forest fires, and transboundary effect.

## 4. Conclusions

In this paper, PM_10_ concentration levels and meteorological parameter data for Mae Hong Son province were collected from 1 January 2009 to 31 December 2017. The results indicated that haze episodes always occurred during the dry season, that is, from February to April. The maximum daily concentration was observed as 309 ppm in March of the year 2012, which was more than twice the ambient air quality standard (with average 42.7 ppm). Factors influencing PM_10_ concentrations included seasonal variation, daily time variation, wind speed and direction, rainfall, and relative humidity. Daily PM_10_ concentration variations produced two peaks. The first peak was observed in the morning, from 7 to 10 am, and the later peak was seen in the afternoon, between 4 to 5 pm However, there was only one peak per day for O_3_ concentrations, from 1 to 3 pm The lowest concentrations of both PM_10_ and O_3_ occurred during the night. The ratios of PM_2.5_ and PM_10_ concentrations onsite in both rural and urban areas in Mae Hong Son were high, being 0.78 and 0.83, respectively. The study suggested a significant negative correlation between relative humidity and PM_10_ (*p*-value < 0.001) while CO was positively correlated to PM_10_ (*p*-value < 0.001). The trajectory model and wind direction in Mae Hong Son during the dry season (from February to April) illustrated that north or northwesterly winds from neighboring countries bring more particulate matter, resulting in the critical haze episode period of each year.

## Figures and Tables

**Table 1:** Statistical records of air pollution and meteorological data in Mae Hong Son Province (2009–2017). PM_10_: particulate matter less than 10 µm in diameter.

Parameter	1 h PM_10_ (ppm)	24 h PM_10_ (ppm)	1 h CO (ppm)	1 h O_3_ (ppb)	Relative Humidity (%)	Temperature (°C)	Pressure (mbar)	Rainfall (mm)
1st Quartile	13.0	15.0	0.40	6.0	57.0	23.0	976.0	0.0
Median	25.0	24.0	0.50	12.0	76.0	25.6	979.0	0.0
Mean	43.0	42.7	0.57	17.6	71.2	25.8	979.5	0.0
3rd Quartile	48.0	47.5	0.70	23.0	88.0	29.6	982.0	0.1
Maximum	726.0	309.2	7.70	123.0	100.0	45.5	994.0	194.0

**Table 2:** Rainfall statistics as recorded at Mae Hong Son Meteorological Station for 2009–2015.

Item	Year						
	2009	2010	2011	2012	2013	2014	2015
Total rain (mm)	841.5	1363.1	1686.1	1475.8	1250.6	1024.9	1064.9
Number of rainy days (days)	132	132	148	144	143	124	127
Daily maximum (mm)	35.6	128.0	99.9	74.8	69.0	55.9	54.2

Source: Meteorological Department, Ministry of Information and Communication Technology [24].
